# Context-dependent ‘safekeeping’ of foraging tools in New Caledonian crows

**DOI:** 10.1098/rspb.2015.0278

**Published:** 2015-06-07

**Authors:** Barbara C. Klump, Jessica E. M. van der Wal, James J. H. St Clair, Christian Rutz

**Affiliations:** Centre for Biological Diversity, School of Biology, University of St Andrews, Sir Harold Mitchell Building, St Andrews KY16 9TH, UK

**Keywords:** corvid, *Corvus moneduloides*, material culture, optimal foraging, tool transportation, tool use

## Abstract

Several animal species use tools for foraging, such as sticks to extract embedded arthropods and honey, or stones to crack open nuts and eggs. While providing access to nutritious foods, these behaviours may incur significant costs, such as the time and energy spent searching for, manufacturing and transporting tools. These costs can be reduced by re-using tools, keeping them safe when not needed. We experimentally investigated what New Caledonian crows do with their tools between successive prey extractions, and whether they express tool ‘safekeeping’ behaviours more often when the costs (foraging at height), or likelihood (handling of demanding prey), of tool loss are high. Birds generally took care of their tools (84% of 176 prey extractions, nine subjects), either trapping them underfoot (74%) or storing them in holes (26%)—behaviours we also observed in the wild (19 cases, four subjects). Moreover, tool-handling behaviour was context-dependent, with subjects: keeping their tools safe significantly more often when foraging at height; and storing tools significantly more often in holes when extracting more demanding prey (under these conditions, foot-trapping proved challenging). In arboreal environments, safekeeping can prevent costly tool losses, removing a potentially important constraint on the evolution of habitual and complex tool behaviour.

## 1. Introduction

Some animal species use tools for foraging [1], such as sticks to extract arthropods and honey from tree cavities [2–6], or stones to break open hard-shelled nuts [3,7], eggs [8] and molluscs [9,10]. While these behaviours provide access to nutritious foods [11,12], they also incur costs in terms of time and energy spent searching for, manufacturing, modifying and deploying tools. One way animals can minimize the costs associated with tool procurement is to use good tools repeatedly, transporting them between foraging sites, and keeping them safe when not needed. Several species have indeed been shown to transport tools for re-use (e.g. chimpanzees [3,13]; orang-utans [14]; capuchin monkeys [15]; sea otters [10]; dolphins [16]; New Caledonian (NC) crows [4,17]; woodpecker finches [18]), but tool ‘safekeeping’ remains poorly documented [13,18]. This is surprising given that such behaviour can enhance the profitability of tool-assisted foraging, with implications for the fitness of individuals (by increasing survival or reproductive success), and ultimately, for evolutionary dynamics.

We investigated the safekeeping of tools in NC crows *Corvus moneduloides*—tropical birds that forage with bill-held tools (for a review, see [19]). Like other avian tool users, NC crows are forced by their anatomy to put down foraging tools in order to process and eat extracted prey items, which inevitably increases the risk of accidental tool loss. Crows indeed occasionally drop tools (for woodpecker finches, see [18]) and can appear notably ‘frustrated’ when this happens (see the electronic supplementary material, movie S2, Scene 1). Importantly, independent anecdotal observations confirm that birds sometimes lodge tools beneath their feet (‘foot-trapping’), or even store them in nearby holes or crevices in-between bouts of probing ([4,20]; see fig. 1G in [21]). A range of factors may affect the costs and likelihood of losing tools, and therefore the relative benefits of safekeeping behaviour, but two aspects of the species' foraging ecology seem particularly important (for further discussion, see §4). First, NC crows use tools in both terrestrial and arboreal settings [4,17], with the costs of dropping tools (in terms of tool recovery) increasing with foraging height. Second, they extract a variety of prey with their tools (for a review, see [19]), including species which can be readily eaten (e.g. small beetle larvae) and others which require prolonged processing (e.g. venomous arthropods, or lizards [21]), with the likelihood of dropping tools presumably increasing with prey-handling demands.

To investigate what NC crows do with their tools between successive prey extractions, we presented wild-caught subjects with extractive foraging opportunities at different heights (‘ground’ and ‘elevated’ conditions), yielding prey which required different handling times (‘easy’ and ‘difficult’ conditions). We found that crows usually took care not to lose their tools and expressed safekeeping behaviour in a context-dependent manner. With growing interest in the ecology and evolutionary origins of animal tool use [19,22–24], recent studies have made good progress with charting the behaviour's energetic benefits (e.g. [10–12,25,26]), but its costs are only rarely investigated (e.g. [11,13,27]). Any attempt to assess the ‘adaptive value’ of tool use requires attention to both, as it relies on comparing the relative profitabilities of tool-use and alternative foraging modes [19]. To our knowledge, our study is the first to examine the behavioural strategies employed by animals to reduce some of the costs associated with tool-assisted foraging. This work raises questions regarding tool procurement, loss and re-use, which can be investigated productively in a wide range of species.

## 2. Material and methods

### (a) Study site and subjects

**Figure 1.**
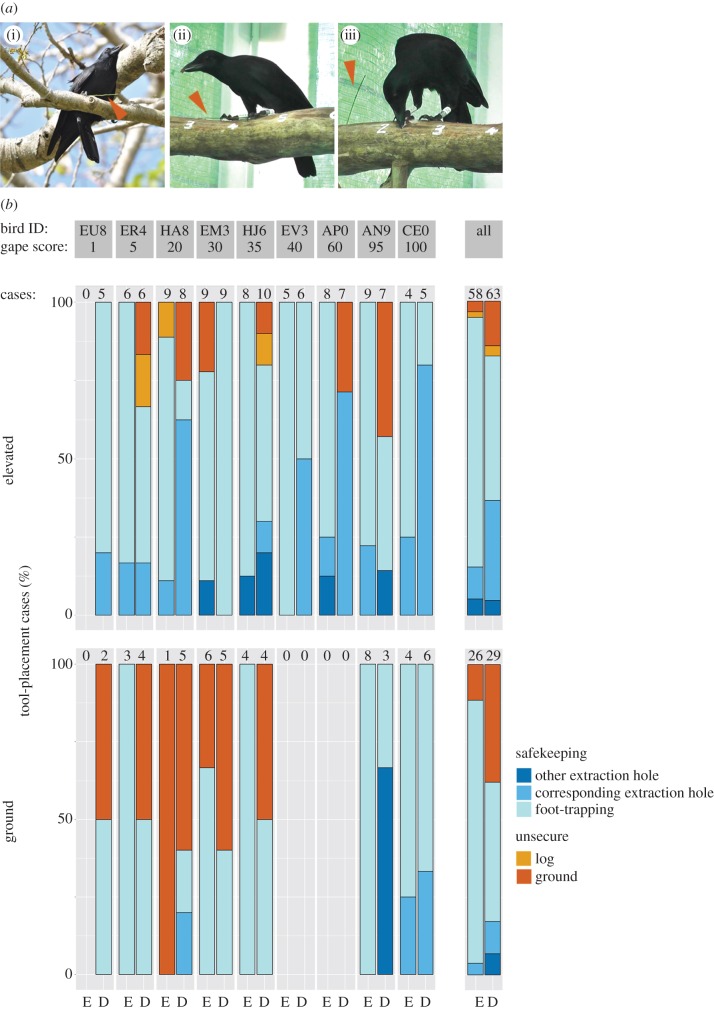
Safekeeping of foraging tools in NC crows. (*a*) Crows employ a range of different safekeeping modes, including foot-trapping ((i) wild bird; (ii) captive bird), and placement in a hole ((iii) captive bird). Tools are marked with red arrows. (*b*) Final tool-placement behaviour (percentage of cases) of nine subjects (identified at the top by their alpha-numerical ring codes) during experiments with two height conditions (‘ground’, bottom; ‘elevated’, top) and two prey-type conditions (‘easy’, E; ‘difficult’, D). Shades of blue indicate secure placement of tools (safekeeping), while orange and red indicate unsecure placement (for definitions, see table 1). Subjects are ordered by gape score (% black coloration; see §2a), a proxy for age and values above bar charts indicate the number of prey extractions, for a given treatment combination, for which tool placement was established (see §2c); the rightmost bars (marked ‘all’) provide summaries across all birds. Each bird participated in two sessions, each consisting of two consecutive trials (for details, see §2b).

Between 24 August and 28 October 2013, we trapped 23 NC crows in a farmland area near Bourail, on the central west coast of New Caledonia, South Pacific. Birds were allocated to age categories based on gape coloration ([12]; the percentage of black coloration increases with age—figure 1), and sexed using morphometric measurements [28]. Three birds were released immediately, as they were breeders, and a fourth one escaped. In pre-testing sessions (for details, see [29]), we identified 13 birds that manufactured and used hooked stick tools and could therefore progress to the main experiment. Of these, one crow was used for pilot-testing, to develop the experimental set-up and refine procedures (see §2b), and three others failed to interact with the final task. Thus, our sample for analyses consisted of five females (two juveniles, two immatures and one adult) and four males (three immatures and one adult).

### (b) Experimental set-up and procedures

Subjects were kept in housing aviaries and tested individually in connected experimental chambers (for details, see [29]). Birds always had access to water and were fed twice a day. Food was removed from housing aviaries either the evening (for morning sessions) or *ca* 1.5 h (afternoon sessions) before testing. Every subject participated in two experimental sessions, each consisting of two consecutive trials: one where a food-baited log (see below) was presented on the ground (ground condition) and another where it was presented on two wooden tripods *ca* 1.30 m above the ground (elevated condition). The order of conditions was randomized across birds (and always different for the two sessions) and did not significantly affect tool-placement behaviour (binary ‘safe’/‘unsecure’ score; generalized linear mixed model (GLMM): *χ*^2^ = 1.84, *p* = 0.18, *n* = 176; for definitions of behaviours, see table 1, and for details of statistical analyses, see §2c).

**Table 1.** Definitions used for scoring the temporary placement of foraging tools in NC crows.

mode	description
(*a*) unsecure	
ground	the tool is lying on the ground, or leaning against the log touching the ground (ground condition only)
log	the tool is lying on the log, not inserted in any hole, and the bird is not touching it
(*b*) safekeeping	
foot-trapping	the bird holds the tool under one or both feet, but is not touching it with its bill (see figure 1*a*(i) and (ii); electronic supplementary material, movie S1, Scenes 5 and 6; movie S2, Scene 2)
corresponding extraction hole	the tool is either left in, or is re-inserted into, the extraction hole from which prey has just been extracted (see electronic supplementary material, movie S1, Scenes 3 and 4)
other extraction hole	the tool is inserted into an extraction hole, other than the one from which prey has just been extracted (see figure 1*a*(iii); electronic supplementary material, movie S1, Scenes 1 and 2); in one case, the tool was wedged into a crevice elsewhere in the aviary

The experimental set-up consisted of two logs. A ‘materials log’ was used to present a single stem of live plant material (from which a tool could be made), firmly wedged into a small hole so that it stood upright. The two stems required for consecutive trials (see above) were visually matched based on diameter and colour, and trimmed to contain only a single fork suitable for hooked stick tool manufacture [30]. By limiting raw materials and forcing crows to ‘pay’ an initial manufacture cost, we attempted to increase the tools' value. A second ‘food log’ contained 10 extraction holes (of 1.6 cm diameter and 7.0 cm depth), each surrounded by eight smaller (4 mm) ‘safekeeping’ holes (which, however, subjects never used for the intended purpose). Each extraction hole was baited with a peanut-sized cube of beef heart (henceforth ‘prey’), which for five of the 10 holes had a downy chicken feather threaded through its centre to increase handling time (difficult condition; e.g. see the electronic supplementary material, movie S1, Scene 1). Allocation of easy (no chicken feather) and difficult prey to the 10 holes was randomized across subjects, but the same for both trials within a session and for both sessions for each bird. In most trials, two tiny pieces of meat were positioned on the food log to attract the birds' attention. One crow (ER4) broke its tool during an elevated trial. This subject had been previously run with slightly different prey preparation (difficult prey was threaded with a chicken feather and a blade of grass) and presentation of tool materials (10 stems rather than a single stem), so we were able to use data from this session instead (no other subjects had experienced these conditions).

An observer filmed experiments with a Panasonic HD camcorder from a hide next to the experimental chamber, and called an assistant via radio if the subject: (i) had not engaged with the set-up for *ca* 15 min; (ii) had not extracted any prey for *ca* 10 min; or (iii) had extracted all prey from the food log. The assistant then removed the food log, tool and any plant debris, and either re-baited the food log (out of the bird's view) and provided a new stem, or after the second trial, concluded the session by opening a passage to the subject's housing aviary. Owing to experimenter error, five trials were terminated prematurely; while this may have reduced the number of prey extractions in these trials, it would not have affected subjects' tool-placement behaviour, so is unproblematic for our analyses.

### (c) Video scoring and statistics

From video, we recorded what type of tool the subject manufactured and used for each extraction. Although most birds made hooked stick tools as intended, some also produced non-hooked stick tools (used in 56 out of 176 instances; see below), or probed with the non-hooked end of a hooked stick tool (a further 13 instances); all data were pooled as there was no significant effect of tool properties on tool-placement behaviour (binary safe/unsecure score; GLMM: *χ*^2^ = 0.17, *p* = 0.68, *n* = 176). We also scored whether prey was extracted, and what the subject did with its tool when not probing with it. After each prey extraction, we recorded the placement of the tool from the moment it was no longer in contact with the bird's bill, until the subject had eaten the prey and picked the tool up again. In cases where the tool was repositioned during this period, we recorded its ‘initial placement’ after extraction as well as its ‘final placement’ (i.e. before its first pick-up following prey consumption). We identified five different types of tool placement, three of which we classed as ‘safekeeping’ and the remainder as ‘unsecure’ (figure 1*b* and table 1).

All videos were scored by B.K. using Solomon Coder (http://solomoncoder.com), and five trials (28%) were re-scored by an independent observer (inter-observer reliability, final safekeeping: *κ* = 0.92); all analyses are based on the original scores. To ensure that all cases were strictly comparable, tool-placement data were excluded from analyses if the subject failed to pick up, or swallow, the prey before extracting prey from another hole, or failed to pick up the tool again before the end of the trial (in total, *n* = 23), as this could indicate reduced levels of motivation. Additionally, cases were excluded when extracted prey had been dropped (*n* = 48; prey-type treatment did not affect the likelihood of prey-dropping; GLMM: *χ*^2^ = 1.44, *p* = 0.23, *n* = 224), as birds in the elevated condition effectively left this experimental condition when they went to the ground to retrieve prey. Our final dataset included 176 initial and 176 final tool-placement scores (figure 1*b*).

We used GLMMs (‘lme4’ package [31] in R [32]) with a binomial error structure and logit-link function to analyse crows' tool-related behaviours, with ‘subject ID’ fitted as a random effect to account for data non-independence. Based on checks with the ‘overdisp_fun( )’ function [33–35], our models were not overdispersed (ratio between the residual deviance and the residual degrees of freedom, *θ* = 0.39–0.97; all *p* > 0.6). Significance of main effects was assessed with likelihood-ratio tests (at *α* = 0.05), and point estimates and 95% CIs are reported on the log-odds scale (see the electronic supplementary material, table S1).

### (d) Observations in the wild

To complement the results from our controlled aviary experiments, we conducted systematic field observations in our study site during the same time period. Some 292 person-hours of fieldwork yielded *ca* 5 h of video recordings, in which five individually identifiable crows (four females, one male; three juveniles and two immatures) performed 25 prey extractions with tools (one to 12 per individual). Five cases could not be scored conclusively owing to poor visibility, and one case was omitted because the bird had dropped its prey (in line with analysis protocols for aviary experiments; see §2c). The remaining 19 cases by four individuals (of which one was also an experimental subject; ER4) were scored by J.v.d.W. as described above (see §2c) and reviewed by B.K., before both observers agreed on consensus classifications.

## 3. Results

### (a) Experiments in captivity

Crows took action to keep their tools safe in the vast majority of cases (84% of 176 extractions, pooled across treatments; figure 1*b*). As predicted, subjects were significantly more likely to express safekeeping behaviour when foraging at height, both for initial (GLMM: *χ*^2^ = 5.80, *p* = 0.02, *n* = 176; Model no. 1 in electronic supplementary material, table S1) and final tool placement (GLMM: *χ*^2^ = 5.56, *p* = 0.02, *n* = 176; Model no. 2; figure 1*b*). While the overall occurrence of foot-trapping (the most common safekeeping mode) was approximately the same at both heights (final tool placement: 64% on the ground versus 62% in the elevated condition), insertion into holes was significantly more frequent in the elevated condition (final tool placement: 11% versus 26%) both for initial (GLMM: *χ*^2^ = 3.96, *p* = 0.05, *n* = 176; Model no. 3) and final tool placement (GLMM: *χ*^2^ = 5.54, *p* = 0.02, *n* = 176; Model no. 4; figure 1*b*). At the trial level, birds dropped tools less frequently (ground versus elevated; mean ± s.d., *n* = 18 cases = 9 subjects × 2 trials; 1.56 ± 2.04 versus 0.78 ± 0.94) and extracted more prey (5.61 ± 3.85 versus 8.17 ± 2.57) when foraging at height.

Prey-handling requirements had a significant effect on final (GLMM: *χ*^2^ = 10.02, *p* = 0.002, *n* = 176; Model no. 6; figure 1*b*), but not on initial (GLMM: *χ*^2^ = 0.07, *p* = 0.79, *n* = 176; Model no. 5), safekeeping behaviour. Against predictions, crows were more likely to express safekeeping when handling easy prey. Closer inspection of the data revealed, however, that crows stored tools in holes significantly more often when handling difficult prey, both with regards to initial (GLMM: *χ*^2^ = 8.19, *p* = 0.004, *n* = 176; Model no. 7) and final tool placement (GLMM: *χ*^2^ = 9.65, *p* = 0.002, *n* = 176; Model no. 8; figure 1*b*)—just as they had done in response to the height treatment (see above). When handling difficult prey, they struggled noticeably with their preferred method of foot-trapping; among initially foot-trapped tools (*n* = 123), 25% were subsequently dropped when handling difficult prey (*n* = 56) compared with only 3% for easy prey (*n* = 67). Only a single initially hole-stored tool was ever dropped (out of a total of *n* = 39 across all treatments), and this occurred when the subject was handling difficult prey.

### (b) Observations in the wild

All observed extractions were made with hooked stick tools, at heights ranging from *ca* 2–10 m. Birds kept their tools safe in all instances, foot-trapping them in the majority of cases (*n* = 15) and storing them in the corresponding extraction hole in the remainder (*n* = 4). Initial and final tool placements were identical in all but two cases (one switch from corresponding extraction hole to foot-trapping; one switch from behind bark (other extraction hole) to foot-trapping).

## 4. Discussion

Our experiments have demonstrated that NC crows' tool-placement behaviour is sensitive to both the costs and likelihood of dropping tools, with certain modes of safekeeping being expressed significantly more often when foraging at height or when handling more demanding prey. Our field observations confirmed that our captive subjects exhibited natural safekeeping behaviours: in both datasets, foot-trapping was the preferred mode (wild: 79% of all cases; elevated condition of experiment: 62%), and short-term storage was more common in extraction holes (wild: 21%; captive: 17%) than in other holes (wild: 0%; captive: 5%).

The costs in terms of time and energy of recovering a dropped tool, or of having to replace it altogether, are expected to increase with foraging height. In our experiments, elevating the food log to only about 1.30 m was sufficient to induce significant changes in subjects' tool-placement behaviour. We suspect that free-ranging crows handle their tools even more cautiously when foraging in the forest canopy tens of metres above ground [4], especially in habitats where dense understory would prevent effective tool retrieval. When foraging on the ground [17], however, placing or dropping tools onto the substrate incurs only small recovery costs, or risk of loss, which may explain both the decreased levels of safekeeping behaviour and the relatively frequent tool-dropping incidents we observed in our experimental ground condition. An alternative explanation is that crows were more nervous on the ground, devoting more attention to vigilance behaviour, and that this increase in ‘cognitive load’ (both activities are demanding; [36–38]) interfered with the normal expression of safekeeping behaviour.

In many habitually tool-using species, tool-related skills are honed during a prolonged developmental period [39–41]. In NC crows, for example, competence in tool manufacture and deployment is attained in the first 1–2 years of life [42,43], during which young birds remain associated with their parents. Safekeeping of tools not only requires considerable motor skills, but is most likely also cognitively demanding, as it necessitates simultaneous attendance to two different stimuli (tool and extracted prey). As dexterity usually increases with age, and proficiency has been shown to decrease attentional demands [44], we would expect experienced adults to outperform younger birds. Although our initial sample of 23 trapped NC crows constituted a substantial proportion of our study population, only nine subjects produced data (see §2a), which unfortunately is insufficient for robust analyses of age effects. Nevertheless, inspection of figure 1*b* suggests that young birds (less than 90% black gape; [12]) indeed performed less safekeeping in the ground condition (57%) than in the elevated condition (89%), while the two adults showed a consistently high level of safekeeping across conditions (ground: 100%; elevated: 88%). A number of factors may contribute to a generally reduced level of safekeeping by young birds. These include insufficient development of the motor skills required to proficiently handle both tool [40,41,45] and prey, and possible cognitive ‘deficiencies', such as an inability to inhibit the primary motivation to eat the prey immediately [46,47]. The fact that the age effect is apparently driven entirely by reduced performance of young birds in the ground condition—with very similar performance of young and adult crows in the elevated condition—is somewhat harder to explain. One possible explanation for this context-dependence is that young subjects may have been relatively more anxious in the ground condition, and thus struggled to attend simultaneously to anti-predator vigilance, prey processing and safekeeping behaviour, while this constraint was alleviated in the elevated condition. Consistent with this, there seems to be a strong effect of age on risk aversion among NC crows in the wild, with young birds often more reluctant than their parents to approach food sources on the ground (such as bait at trap sites), preferring instead to beg from the safety of the canopy.

Age does not, however, explain the observed context-dependence of safekeeping modes, in which storage in holes formed a larger proportion of safekeeping events at height than it did on the ground. In fact, of the nine subjects, only three employed this mode on the ground, while all of them did on the elevated log (figure 1*b*). This pattern suggests that safekeeping modes may differ in the relative level of security they afford: placing a tool in a hole clearly reduces the risk of dropping, while foot-trapping seems to be less secure, especially when handling prey that requires additional processing before it can be eaten (see below). But, accidental dropping is not the only way that crows can lose tools in the wild; we have repeatedly observed wild birds picking up tools that other individuals had just left or placed in holes [42]. This highlights an interesting dilemma crows may face: inserting tools into holes is better for preventing accidental loss, but increases the likelihood of ‘tool kleptoparasitism’, while the opposite holds true for foot-trapping. On the ground, but not at height, the cost of dropping tools is minimal (see above), which may explain our finding of height-dependent changes in safekeeping modes.

Further fieldwork is required to examine whether the proximity of conspecifics influences the tool-placement behaviour of foraging NC crows, and if so, whether this holds true only for encounters between unrelated birds or also for close kin [48]. We expect that the risk of tool kleptoparasitism is widespread among tool-using species. In primates, for example, tool-assisted foraging usually takes place in social groups [13,15], which creates opportunities for close-range interaction and tool stealing [13,49]. The scarcity of reports of tool kleptoparasitism may be due to the fact that, so far, researchers simply have not paid much attention to this phenomenon, but it could also indicate that animals employ very effective countermeasures (the same way that predation events are rarely observed, because prey exhibit effective anti-predator strategies; see [36]). Apart from the safekeeping modes examined in our study of NC crows (foot-trapping and storage in holes and crevices), animals may use their bodies to shield tools from conspecifics, or even actively defend tools that are not being used. In general, we predict tool-safekeeping behaviours to be sensitive to the costs associated with tool procurement and manufacture (see §1), and thus tool value. The investigation of tool safekeeping as an anti-kleptoparasitism strategy clearly offers valuable opportunities for comparative analyses.

As hypothesized, tool-placement behaviour varied with prey-handling requirements in our experiments, but surprisingly, crows performed safekeeping behaviours more often when handling easy prey (figure 1*b*). This could be due to the fact that birds often struggled with the handling of difficult prey and consequently failed to secure their tools. The removal of the feather from the meat cube seemed challenging (see the electronic supplementary material, movie S1, Scene 6; although some birds swallowed the prey with the feather—see the electronic supplementary material, movie S1, Scene 3), and tools which had initially been securely trapped under the foot sometimes dropped to the ground as the subject focused its attention on the food item (for further discussion of attentional demands, see above). While storage in holes kept tools safe during the processing of both easy and difficult prey, birds that initially foot-trapped tools succeeded in keeping them safe when handling easy prey, but struggled to do so when handling difficult prey. Importantly, final storage in holes was significantly more common when subjects handled difficult prey, an effect resembling that observed for the height treatment.

While we specifically investigated the short-term storage of tools (for seconds to minutes), our results encourage work on tool ‘caching’ over longer time periods (in the order of hours to days). Some animals have been shown to anticipate future needs and plan ahead (for reviews, see [50,51]), an ability long thought to be uniquely human [52]. In a tool-using context, it has recently been shown that untrained orang-utans and bonobos [53], but not long-tailed macaques [54], transport tools in anticipation of future food extractions. The storage of tools in NC crows over comparable timescales (several hours) remains to be demonstrated, but would be expected, given that the species is known to cache food [45,55], and food caching and tool use show striking ontogenetic parallels [56]. The fact that our subjects anticipated that they would need their tools again, and took context-appropriate precautions to keep them safe, demonstrates a form of ‘planning’ that may substantially increase the efficiency of tool use. Our experiment was designed to elucidate the behavioural ecology of crows' tool-handling behaviour, rather than the cognitive mechanisms involved. This said, we believe that our novel experimental paradigm will prove useful in future studies that wish to probe the cognitive processes of short- and long-term planning.

Tool use has been studied intensively in a range of species [1], but the safekeeping of tools between successive foraging episodes remains poorly documented. As explored above, the costs of dropping tools are expected to increase with foraging height. NC crows aside ([4,20,21]; this study), there are anecdotal observations of woodpecker finches storing foraging tools temporarily in a crevice or under bark [18], and of chimpanzees placing tools in forked branches, or between their toes, when ant-dipping in the forest canopy [13]. In fact, several primate species use foraging tools in arboreal habitats: for example, chimpanzees hunt bush babies [57], and dip for ants [13] and honey [3]; blonde capuchins fish for termites [6]; and orang-utans extract honey [5], and feed with mouth-held tools on *Neesia* spp. fruit, often processing multiple fruit sequentially [14]. In all of these cases, tool safekeeping has the potential to increase foraging efficiency, by avoiding the costly retrieval, or successive sourcing, of good tools. Safekeeping behaviours are probably easily overlooked, as they can be surprisingly swift and subtle (see the electronic supplementary material, movies S1 and S2), especially when expressed by skilled adults, and fieldworkers tend to focus on details of tool manufacture or prey extraction. There is considerable scope for comparative work on animals' tool safekeeping behaviour, both through observing free-ranging subjects, and through controlled experimentation in captivity.

It has recently been suggested that habitual and complex tool use (i.e. plasticity in making and using tools) is less likely to evolve when tool-use opportunities occur in arboreal environments, where rates of innovation (range of tool materials), social learning (visibility) and accumulation (access to previously manufactured tools) may be lower than in terrestrial settings ([58]; for similar arguments for aquatic environments, see [59]). Strategies to keep successful tools safe can increase foraging profitability, potentially removing some of the constraints experienced by arboreal tool users.

## Supplementary Material

Table S1

## Data Accessibility

Raw data are shown in figure 1.

## References

[1] Shumaker RW Walkup KR Beck BB 2011 Animal tool behavior: the use and manufacture of tools by animals. Baltimore, MD: John Hopkins University Press.

[2] Eibl-Eibesfeldt I 1961 Über den Werkzeuggebrauch des Spechtfinken *Camarhynchus pallidus*. Z. Tierpsychol. 18, 343–346. (10.1111/j.1439-0310.1961.tb00424.x)

[3] McGrew WC 1992 Chimpanzee material culture. Cambridge, UK: Cambridge University Press.

[4] Hunt GR 1996 Manufacture and use of hook-tools by New Caledonian crows. Nature 379, 249–251. (10.1038/379249a0)

[5] van Schaik CP 2009 Orangutan cultures revisited. In Orangutans: geographic variation in behavioral ecology and conservation (eds Wich SA Utami SS Setia TM van Schaik CP), pp. 299–309. New York, NY: Oxford University Press.

[6] Souto A Bione CBC Bastos M Bezerra BM Fragaszy D Schiel N 2011 Critically endangered blonde capuchins fish for termites and use new techniques to accomplish the task. Biol. Lett. 7, 532–535. (10.1098/rsbl.2011.0034) 21389018 PMC3130233

[7] Fragaszy D Izar P Visalberghi E Ottoni EB de Oliveira MG 2004 Wild capuchin monkeys (*Cebus libidinosus*) use anvils and stone pounding tools. Am. J. Primatol. 64, 359–366. (10.1002/ajp.20085) 15580579

[8] Van Lawick-Goodall J Van Lawick-Goodall H 1966 Use of tools by the Egyptian vulture, *Neophron percnopterus*. Nature 212, 1468–1469. (10.1038/2121468a0)

[9] Malaivijitnond S Lekprayoon C Tandavanittj N Panha S Cheewatham C Hamada Y 2007 Stone-tool usage by Thai long-tailed macaques (*Macaca fascicularis*). Am. J. Primatol. 69, 227–233. (10.1002/ajp.20342) 17146796

[10] Fujii JA Ralls K Tinker MT 2014 Ecological drivers of variation in tool-use frequency across sea otter populations. Behav. Ecol. 26, 519–526. (10.1093/beheco/aru220)

[11] Tebbich S Taborsky M Fessl B Dvorak M 2002 The ecology of tool-use in the woodpecker finch (*Cactospiza pallida*). Ecol. Lett. 5, 656–664. (10.1046/j.1461-0248.2002.00370.x)

[12] Rutz C Bluff LA Reed N Troscianko J Newton J Inger R Kacelnik A Bearhop S 2010 The ecological significance of tool use in New Caledonian crows. Science 329, 1523–1526. (10.1126/science.1192053) 20847272

[13] Nishida T Hiraiwa M 1982 Natural history of a tool-using behavior by wild chimpanzees in feeding upon wood-boring ants. J. Hum. Evol. 11, 73–99. (10.1016/S0047-2484(82)80033-X)

[14] Van Schaik CP Knott CD 2001 Geographic variation in tool use on *Neesia* fruits in orangutans. Am. J. Phys. Anthropol. 114, 331–342. (10.1002/ajpa.1045) 11275962

[15] Ottoni EB Izar P 2008 Capuchin monkey tool use: overview and implications. Evol. Anthropol. Issues News Rev. 17, 171–178. (10.1002/evan.20185)

[16] Smolker R Richards A Connor R Mann J Berggren P 1997 Sponge carrying by dolphins (Delphinidae, *Tursiops* sp.): a foraging specialization involving tool use? Ethology 103, 454–465. (10.1111/j.1439-0310.1997.tb00160.x)

[17] Rutz C Bluff LA Weir AAS Kacelnik A 2007 Video cameras on wild birds. Science 318, 765 (10.1126/science.1146788) 17916693

[18] Tebbich S Teschke I Cartmill EA Stankewitz S 2012 Use of a barbed tool by an adult and a juvenile woodpecker finch (*Cactospiza pallida*). Behav. Process. 89, 166–171. (10.1016/j.beproc.2011.10.016) 22085790

[19] Rutz C St Clair JJH 2012 The evolutionary origins and ecological context of tool use in New Caledonian crows. Behav. Process. 89, 153–165. (10.1016/j.beproc.2011.11.005) 22209954

[20] Orenstein RI 1972 Tool-use by the New Caledonian crow (*Corvus moneduloides*). Auk 89, 674–676.

[21] Troscianko J Bluff LA Rutz C 2008 Grass-stem tool use in New Caledonian crows *Corvus moneduloides*. Ardea 96, 283–285. (10.5253/078.096.0214)

[22] Biro D Haslam M Rutz C 2013 Tool use as adaptation. Phil. Trans. R. Soc. B 368, 20120408 (10.1098/rstb.2012.0408) 24101619 PMC4027410

[23] Sanz C Call J Boesch C 2013 Tool use in animals: cognition and ecology. New York, NY: Cambridge University Press.

[24] Koops K Visalberghi E van Schaik CP 2014 The ecology of primate material culture. Biol. Lett. 10, 20140508 (10.1098/rsbl.2014.0508) 25392310 PMC4261853

[25] Yamakoshi G 1998 Dietary responses to fruit scarcity of wild chimpanzees at Bossou, Guinea: possible implications for ecological importance of tool use. Am. J. Phys. Anthropol. 106, 283–295. (10.1002/(SICI)1096-8644(199807)106:3<283::AID-AJPA2>3.0.CO;2-O) 9696145

[26] Sanz CM Morgan DB 2013 Ecological and social correlates of chimpanzee tool use. Phil. Trans. R. Soc. B 368, 20120416 (10.1098/rstb.2012.0416) 24101626 PMC4027411

[27] Mann J Sargeant BL Watson-Capps JJ Gibson QA Heithaus MR Connor RC Patterson E 2008 Why do dolphins carry sponges? PLoS ONE 3, e3868 (10.1371/journal.pone.0003868) 19066625 PMC2587914

[28] Kenward B Rutz C Weir AAS Chappell J Kacelnik A 2004 Morphology and sexual dimorphism of the New Caledonian crow *Corvus moneduloides*, with notes on its behaviour and ecology. Ibis 146, 652–660. (10.1111/j.1474-919x.2004.00299.x)

[29] St Clair JJH Rutz C 2013 New Caledonian crows attend to multiple functional properties of complex tools. Phil. Trans. R. Soc. B 368, 20120415 (10.1098/rstb.2012.0415) 24101625 PMC4027419

[30] Hunt GR Gray RD 2004 The crafting of hook tools by wild New Caledonian crows. Biol. Lett. 271 (Suppl. 3), S88–S90. (10.1098/rsbl.2003.0085) PMC1809970 15101428

[31] Bates D Maechler M Bolker B Walker S 2014 Lme4: linear mixed-effects models using Eigen and S4. In R package v. 1.1-6. See http://CRAN.R-project.org/package=lme4.

[32] R Core Team. 2014 R: a language and environment for statistical computing. Vienna, Austria: R Foundation for Statistical Computing.

[33] Doran C Pearce T Connor A Schlegel T Franklin E Sendova-Franks AB Franks NR 2013 *Economic investment by ant colonies in searches for better homes*. Biol. Lett. 9, 20130685 (10.1098/rsbl.2013.0685) 24088565 PMC3971717

[34] Baldacchino F Puech L Manon S Hertzog LR Jay-Robert P 2014 Biting behaviour of Tabanidae on cattle in mountainous summer pastures, Pyrenees, France, and effects of weather variables. Bull. Entomol. Res. 104, 471–479. (10.1017/S0007485314000170) 24622151

[35] Fitzpatrick JL Evans JP 2014 Postcopulatory inbreeding avoidance in guppies. J. Evol. Biol. 27, 2585–2594. (10.1111/jeb.12545) 25387854

[36] Lima SL Dill LM 1990 Behavioral decisions made under the risk of predation: a review and prospectus. Can. J. Zool. 68, 619–640. (10.1139/z90-092)

[37] Clark CW Dukas R 2003 The behavioral ecology of a cognitive constraint: limited attention. Behav. Ecol. 14, 151–156. (10.1093/beheco/14.2.151)

[38] Seed AM Call J Emery NJ Clayton NS 2009 Chimpanzees solve the trap problem when the confound of tool-use is removed. J. Exp. Psychol. Anim. Behav. Process. 35, 23–34. (10.1037/a0012925) 19159160

[39] Meulman EJM Seed AM Mann J 2013 If at first you don't succeed… studies of ontogeny shed light on the cognitive demands of habitual tool use. Phil. Trans. R. Soc. B 368, 20130050 (10.1098/rstb.2013.0050) 24101632 PMC4027412

[40] Lonsdorf E 2006 What is the role of mothers in the acquisition of termite-fishing behaviors in wild chimpanzees (*Pan troglodytes schweinfurthii*)? Anim. Cogn. 9, 36–46. (10.1007/s10071-005-0002-7) 16195914

[41] De Resende BD Ottoni EB Fragaszy DM 2008 Ontogeny of manipulative behavior and nut-cracking in young tufted capuchin monkeys (*Cebus apella*): a perception–action perspective. Dev. Sci. 11, 828–840. (10.1111/j.1467-7687.2008.00731.x) 19046151

[42] Bluff LA Troscianko J Weir AAS Kacelnik A Rutz C 2010 Tool use by wild New Caledonian crows *Corvus moneduloides* at natural foraging sites. Proc. R. Soc. B 277, 1377–1385. (10.1098/rspb.2009.1953) PMC2871937 20053646

[43] Holzhaider J Hunt G Gray R 2010 The development of *Pandanus* tool manufacture in wild New Caledonian crows. Behaviour 147, 553–586. (10.1163/000579510X12629536366284)

[44] Dukas R 2002 Behavioural and ecological consequences of limited attention. Phil. Trans. R. Soc. Lond. B 357, 1539–1547. (10.1098/rstb.2002.1063) 12495511 PMC1693070

[45] Kenward B Rutz C Weir AAS Kacelnik A 2006 Development of tool use in New Caledonian crows: inherited action patterns and social influences. Anim. Behav. 72, 1329–1343. (10.1016/j.anbehav.2006.04.007)

[46] Amici F Aureli F Call J 2008 Fission–fusion dynamics, behavioral flexibility, and inhibitory control in primates. Curr. Biol. 18, 1415–1419. (10.1016/j.cub.2008.08.020) 18804375

[47] Call J 2010 Trapping the minds of apes: causal knowledge and inferential reasoning about object-object interactions. In The mind of the chimpanzee (eds Lonsdorf E Ross SR Matsuzawa T), pp. 75–86. Chicago, IL: The University of Chicago press.

[48] Rutz C Burns ZT James R Ismar SM Burt J Otis B Bowen J St Clair JJH 2012 Automated mapping of social networks in wild birds. Curr. Biol. 22, R669–R671. (10.1016/j.cub.2012.06.037) 22974988

[49] Matsuzawa T 1999 Communication and tool use in chimpanzees: cultural and social contexts. In The design of animal communication (eds Hauser MD Konishi M), pp. 645–672. Cambridge, MA: The MIT Press.

[50] Roberts WA 2007 Mental time travel: animals anticipate the future. Curr. Biol. 17, R418–R420. (10.1016/j.cub.2007.04.010) 17550769

[51] Roberts WA 2012 Evidence for future cognition in animals. Learn. Motiv. 43, 169–180. (10.1016/j.lmot.2012.05.005)

[52] Tulving E 1984 Précis of elements of episodic memory. Behav. Brain Sci. 7, 223–238. (10.1017/S0140525X0004440X)

[53] Mulcahy NJ Call J 2006 Apes save tools for future use. Science 312, 1038–1040. (10.1126/science.1125456) 16709782

[54] Dekleva M van den Berg L Spruijt BM Sterck EHM 2012 Take it or leave it: transport of tools for future use by long-tailed macaques (*Macaca fascicularis*). Behav. Process. 90, 392–401. (10.1016/j.beproc.2012.04.003) 22579441

[55] Hunt G 2000 Tool use by the New Caledonian crow *Corvus moneduloides* to obtain cerambycidae from dead wood. EMU 100, 109–114. (10.1071/MU9852)

[56] Kenward B Schloegl C Rutz C Weir AAS Bugnyar T Kacelnik A 2011 On the evolutionary and ontogenetic origins of tool-oriented behaviour in New Caledonian crows (*Corvus moneduloides*). Biol. J. Linn. Soc. 102, 870–877. (10.1111/j.1095-8312.2011.01613.x) PMC4398869 25892825

[57] Pruetz JD Bertolani P 2007 Savanna chimpanzees, *Pan troglodytes* *verus*, hunt with tools. Curr. Biol. 17, 412–417. (10.1016/j.cub.2006.12.042) 17320393

[58] Meulman EJM Sanz CM Visalberghi E van Schaik CP 2012 The role of terrestriality in promoting primate technology. Evol. Anthropol. Issues News Rev. 21, 58–68. (10.1002/evan.21304) 22499440

[59] Mann J Patterson EM 2013 Tool use by aquatic animals. Phil. Trans. R. Soc. B 368, 20120424 (10.1098/rstb.2012.0424) 24101631 PMC4027413

