# Supplementary material for: Context-dependent ‘safekeeping’ of foraging tools in New Caledonian crows
Source: Proc Biol Sci. 2015 Jun 7;282(1808):20150278. doi: 10.1098/rspb.2015.0278 (PMC4455803; doi:10.1098/rspb.2015.0278)
Supplement: Table S1 [file rspb20150278supp1.pdf]

Table S1. Point estimates* and lower and upper limits of 95% confidence intervals (CI) (all on the log-odds scale) for the eight models reported in the Results section of the main text (§3). All response and explanatory variables were binary, and scored as follows: tool placement ('safe' and 'unsecure') and storage in holes ('yes' and 'no'). The conditions refer to two experimental treatments, as described in the Methods section (§2): height ('ground' and 'elevated') and prey type ('easy' and 'difficult').

**Model**		**Condition**	**Lower limit for 95% CI**	**Point estimate**	**Upper limit for 95% CI**
#1	Initial tool placement ~ height + (1|BirdID)	Intercept (Elevated)	1.58	4.79	7.99
		Ground	-2.78	-1.52	-0.26
#2	Final tool placement ~ height + (1|BirdID)	Intercept (Elevated)	1.45	2.16	2.87
		Ground	-1.90	-1.04	-0.18
#3	Initial storage in holes ~ height + (1|BirdID)	Intercept (Elevated)	-1.70	-1.09	-0.47
		Ground	-1.85	0.91	0.02
#4	Final storage in holes ~ height + (1|BirdID)	Intercept (Elevated)	-1.68	-1.07	-0.47
		Ground	-2.10	-1.11	-0.13
#5	Initial tool placement ~ prey type + (1|BirdID)	Intercept (Easy)	1.06	4.14	7.22
		Difficult	-1.34	-0.16	1.02
#6	Final tool placement ~ prey type + (1|BirdID)	Intercept (Easy)	1.74	2.70	3.66
		Difficult	-2.42	-1.45	-0.48
#7	Initial storage in holes ~ prey type + (1|BirdID)	Intercept (Easy)	-2.85	-2.02	1.18
		Difficult	0.33	1.14	1.95
#8	Final storage in holes ~ prey type + (1|BirdID)	Intercept (Easy)	-2.98	-2.12	1.27
		Difficult	0.43	1.26	2.09

*Point estimates of the intercepts are the log-odds of getting a 1 in the response variable when the bird is at height (Intercept Elevated) or handling easy prey (Intercept Easy). The second point estimate (Ground or Difficult) describes the change in log-odds (to get a 1 in the response variable) from the point estimate of the intercepts when the bird is on the ground or handling difficult prey.
